# Toxic Leukoencephalopathy After “Chasing the Dragon” With a Non-Heroin Opioid

**DOI:** 10.7759/cureus.45774

**Published:** 2023-09-22

**Authors:** Aayushi Agarwal, Megan Evans, Raja Mogallapu, Nycole Kothe, Michael Ang-Rabanes

**Affiliations:** 1 Psychiatry, West Virginia University School of Medicine, Martinsburg, USA

**Keywords:** leukoencephalopathy, opioids, chasing the dragon, oxycodone, fentanyl

## Abstract

In the United States, the rate of opioid use increases each year. With this, users are engaging in more non-traditional methods of usage. “Chasing the dragon” is a term used to describe opioid inhalation, where the user heats the opioid and then inhales the smoke. While this method of usage is typically associated with a quicker high and fewer adverse effects, it can also lead to toxic leukoencephalopathy (TLE). TLE is defined as a structural alteration of the brain’s white matter due to toxic exposure, such as heroin. A 57-year-old woman with a history of polysubstance abuse was admitted to the hospital after weeks of erratic behavior. At presentation, her urine drug screen was found to be positive for oxycodone (which was prescribed to her) and fentanyl. A brain MRI was eventually done, which showed a periventricular leukoencephalopathy characteristic of opioid inhalation. Traditionally, opioid-related TLE is due to heroin, and patients are found to have very dramatic motor issues. As this patient did not report a history of heroin use and did not present with significant motor deficits, this report highlights the need to maintain a level of suspicion for TLE. As levels of opioid use continue to rise, it is likely that many presentations like that of the patient outlined in this report will be seen.

## Introduction

West Virginia has long been the epicenter of opioid usage and overdoses. Nationally, opioid overdose deaths surpassed 100,000 during a 12-month period ending in June 2021 [1]. During this time, most opioid-related deaths in both West Virginia and nationally were attributed to the synthetic opioid, fentanyl [1,2,3]. From 2005 to 2015, the rate of opioid users who inject themselves increased by nearly 20%, from 36% to 54% [4]. Although not specifically explored in West Virginia, the rate of opioid use through snorting or smoking has likely also seen an uptick in the state. Snorting or smoking opioids can lead to toxic leukoencephalopathy (TLE), a rare condition marked by damage to the brain's white matter. This condition is often linked to a practice called "chasing the dragon," where users heat heroin on aluminum foil and inhale the smoke through a straw [5,6,7,8]. TLE usually presents with motor symptoms developing over weeks to months [9]. This report describes the presentation and outcome of a patient suffering from opioid-induced TLE, primarily characterized by behavioral abnormalities and delirium.

## Case presentation

**Figure 1:**
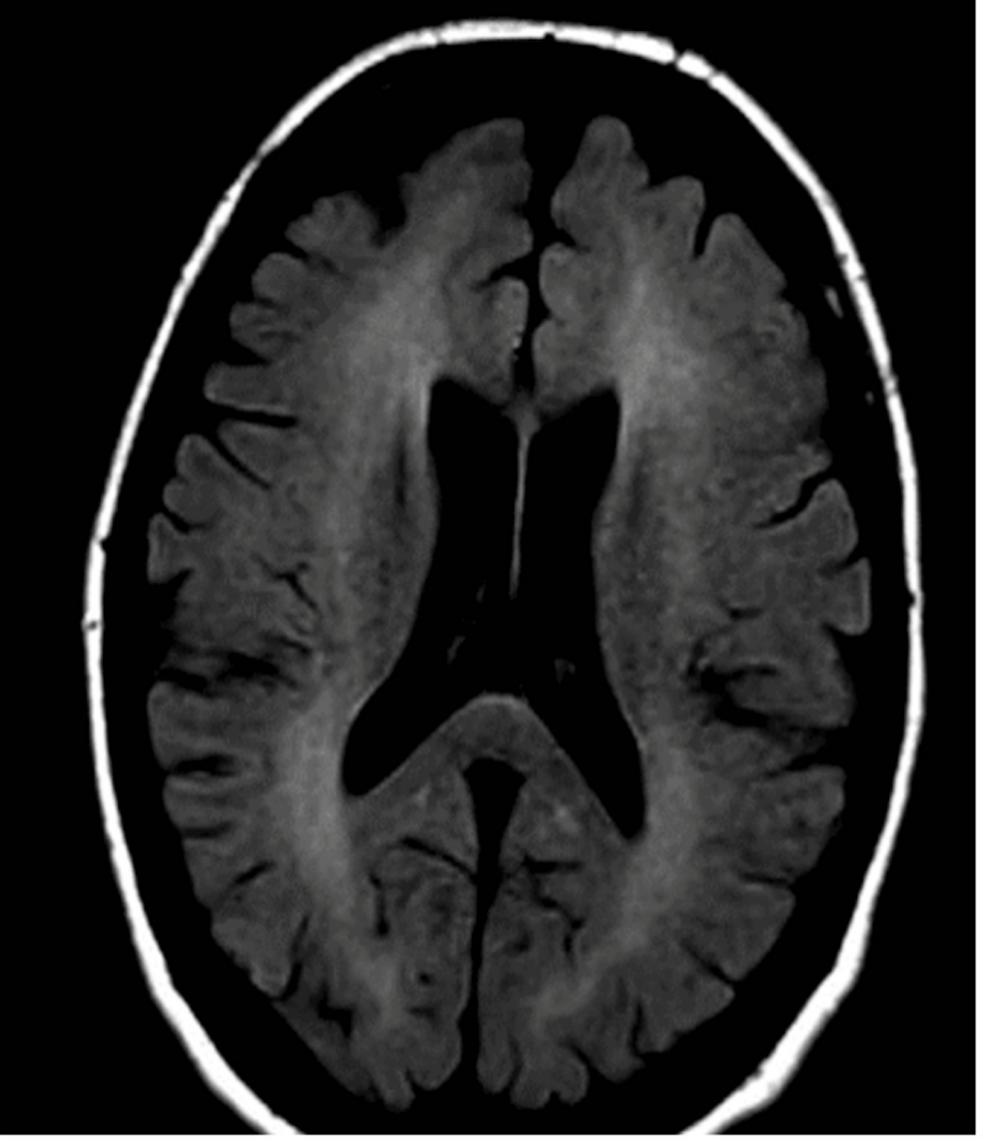
Brain MRI showing extensive periventricular leukoencephalopathy, characteristic for opioid inhalation.

A 57-year-old woman with a history of polysubstance use was admitted to the psychiatric unit after being found unclothed and confused. At the time of admission, the patient was aggressive, paranoid, illogical, disorganized, and hyperverbal. Her mini mental status exam (MMSE) score was 12/30. Per her family, she had been acting increasingly erratic for weeks with few motor symptoms. Her qualitative urine drug screen was positive for fentanyl and oxycodone and negative for hepatitis C and HIV. Throughout the 26-day hospitalization, she adamantly denied having ever used any drugs other than her prescribed oxycodone and a singular instance in which she stated her sister "blew cocaine up [her] nose." Despite this, multiple puncture scars on her arms and hands were seen on physical exam, likely relating to IV drug use. She was ultimately diagnosed with substance-induced psychosis with suspicion of opioid inhalation ("chasing the dragon"). The patient was also hospitalized in the ICU for two days at the same facility a month prior to this hospitalization after being found unresponsive at home. During that stay, a lumbar puncture showed elevated glucose and proteins in her cerebrospinal fluid. At the time, the patient was diagnosed with acute renal failure and possible microbial infection, likely secondary due to polysubstance overdose and aspiration. Her qualitative urine drug screen at that admission was positive for polysubstance use. For the first few weeks of hospitalization, the patient had an ataxic gait, confusion, and was unoriented to time and situation. The patient was tried on multiple antipsychotic medications and slightly improved with quetiapine. The neurological team was consulted and agreed that her presentation was consistent with an acute encephalopathy related to opioid inhalation. This diagnosis was confirmed later in the hospitalization when the patient consented to an MRI (Figure 1). An EEG was also conducted during her stay, revealing nonspecific findings but no seizures or epileptiform discharges. CT scans of her brain conducted throughout her admission were consistent, showing no acute abnormalities. Initially, the patient exhibited elevated serum ammonia levels, a possible cause for the encephalopathy. However, elevated levels of valproic acid were also present. Consequently, Depakote was discontinued, and the levels were monitored until they returned to normal.

Towards the end of her hospitalization, her delirium and confusion began to improve, with her MMSE score rising from 12/30 to 23/30. However, she started to confabulate about the duration of her hospital stay and the reason for her admission, replacing previous points of confusion (the patient did not have a significant history of alcohol use). Attempts to initiate anti-dementia medications were unsuccessful due to the patient's non-compliance. Given the severity of the case, a full return to her baseline is improbable, leading to her eventual discharge.

## Discussion

Leukoencephalopathy is the overarching term for white matter diseases of varying causes associated with behavioral changes, ataxia, and cognitive defects. Leukoencephalopathy, as a diagnosis, is nonspecific and can be associated with various autoimmune diseases, chromosomal disorders, structural defects, and external factors, such as ingestion of toxins [10]. TLE can be caused by chemotherapeutic drugs, environmental toxins, and, in many cases, substance use. The degree of deficit in patients depends on a multitude of factors, including dosages and length of exposure to toxins, level of toxicity, and patient history [10,11]. The use of heroin via snorting or smoking is a common cause of TLE, leading to the accumulation of opioids in the myelin sheath [12]. "Chasing the dragon" is the process of smoking various illicit and prescription drugs, commonly heroin or other opioids. Here, the drug is placed on a piece of aluminum foil, heated with a flame, and the resulting vapor is inhaled through a tube [6]. Many choose to use this route of administration to avoid the systemic adverse effects associated with injection and oral opioid use while also increasing the euphoric effects due to the vapors immediately reaching the brain [13]. Another common use of the expression "chasing the dragon" refers to the ongoing, futile pursuit of the euphoria, or high, that a person experiences the first time they use a drug, leading to a cycle of continued use and addiction. The usage of opioids specifically has a lot to do with their biochemistry. Opioid receptors are G-protein coupled receptors that act in the cAMP/protein kinase A pathway [8]. The main clinical effect of opioids is analgesia, which is caused by binding to the "mu," "kappa," and "delta" receptors. The euphoric feeling, or "high," that comes from opioids is due to the downstream GABA inhibition after binding to the "mu" receptor, causing an excess of dopamine. However, this exact mechanism leads to respiratory depression, constipation, euphoria, dysphoria, and dependence that can occur with continued use [14].

Heroin-induced TLE manifests in three stages: ataxia, softened speech and motor restlessness, and myoclonic jerks and spastic paresis. The final stage, characterized by spasms and hypotonic paresis, can be fatal [11,13,15]. This case reveals a rare instance of TLE following the suspected inhalation of opioids, specifically fentanyl and oxycodone. Due to her altered mental status, the patient, who is a poor historian, reported no history of heroin use; however, this cannot be confirmed, constituting a limitation of this report. Although the exact route of use is unknown due to the patient's denial of substance use, snorting and inhalation were suspected due to clinical signs. While substance use-related TLE is normally due to heroin, this patient's urine drug screening (UDS) was positive only for oxycodone and fentanyl. Additionally, the patient's prior history of cocaine use could play a role in her presentation. Research shows that the cause of cocaine-induced TLE is often not the cocaine itself but rather due to additives in the cocaine that is ingested [16]. Beyond etiology, this case is unique due to the patient's presentation: her primary symptom was altered mental status rather than ataxia. While ataxia was exhibited at the beginning of her hospitalization, it was very mild compared to other previously documented cases of TLE. Additionally, the patient began confabulating during the interview, which is not typically observed. Cerebellar involvement, as seen with the patient's ataxia, is present in both heroin and oxycodone-induced encephalopathy. Heroin has an increased affinity for "mu" receptors, whereas oxycodone has an affinity for "kappa" receptors, both of which are found in the cerebellum [12]. Oxycodone specifically has a very high oral bioavailability, which allows for a high plasma concentration rather quickly. This can trigger side effects like cerebellar ataxia and cognitive changes within hours [12]. At present, most non-prescription opioids are laced with fentanyl or composed almost entirely of it. Fentanyl has an exceptionally high affinity for 'mu' receptors - significantly more than heroin [17]. Therefore, the presentation of fentanyl-induced TLE is likely very similar to that of heroin-induced TLE. Presentation of substance-induced TLE can vary significantly among patients, making it a difficult clinical diagnosis. Leukoencephalopathy can present in both reversible and irreversible forms and can present with behavioral changes, as detailed in this case report. Unfortunately, little is known about the pathogenesis of TLE, as well as treatments beyond the cessation of exposed toxins [18].

## Conclusions

This case is specifically important due to its presentation and etiology. This patient's condition was primarily described as delirium and behavioral changes with only mild ataxia. Additionally, the etiology of this patient's condition is likely related to fentanyl (or oxycodone) rather than heroin inhalation, despite heroin being the most reported cause. While this patient may have believed she was using heroin, it is essential to document this case as fentanyl-induced TLE, which may be seen more frequently in the future. Recognizing and understanding the variances in clinical presentation can allow for improvement in clinical outcomes for patients with TLE due to inhalation of drugs. Specifically, this case highlights the necessity to maintain suspicion for TLE in all patients with altered mental status and a history of drug use, with or without heroin use. In states like West Virginia, the number of people who use and overdose on opioids continues to rise yearly, leading to a likely increase in case presentations similar to this patient's. As this patient will likely never return to baseline due to her extensive history of polysubstance use and the severity of her symptoms, this case emphasizes the need for future research into treatment and prevention of TLE.
